# Clinical outcomes of double-screw fixation with bone grafting for displaced scaphoid nonunions: A series of 21 cases

**DOI:** 10.3389/fsurg.2023.1096684

**Published:** 2023-02-17

**Authors:** Wei Ma, Jeffrey Yao, Yang Guo

**Affiliations:** ^1^ Department of Orthopedic Surgery, Air Force Medical Center, Beijing, China; ^2^ Department of Orthopedic Surgery, Stanford University Medical Center, Redwood City, CA, United States; ^3^ Department of Hand Surgery, Beijing Jishuitan Hospital, The Fourth Clinical College of Peking University, Beijing, China

**Keywords:** displaced scaphoid nonunion, double screws fixation, bone grafting, clinical outcome, humpback deformity

## Abstract

**Purpose:**

This study reports the clinical outcomes of double-screw fixation with bone grafting for displaced scaphoid nonunions.

**Patients and methods:**

This study was a retrospective survey. From January 2018 to December 2019, 21 patients with displaced scaphoid fractures underwent open debridement and two headless compression screw fixation with bone grafting. The preoperative and postoperative lateral intrascaphoid angle (LISA) and scapholunate angle (SLA) were recorded. Preoperative and postoperative grip strength (% of the healthy side), active range of motion (AROM), visual analogue scale (VAS), and patient-rated wrist evaluation (PRWE) scores at the final follow-up were obtained for all patients for comparison.

**Results:**

Patients were treated for an average of 38.3 months (range 12–250) after the injury. The average time of postoperative follow-up was 30.5 months (range 24–48). All fractures achieved union at a mean of 2.7 months (range 2–4) after surgery, and 14 scaphoids of 21 patients (66.7%) healed by 8 weeks. CT scans showed no evidence of cortical penetration of either screw in all patients. There was a statistically significant improvement in AROM, grip strength, and PRWE. No complications occurred in this study, and all patients returned to work.

**Conclusion:**

This study indicates that double-screw fixation with bone grafting is an effective technique for treating displaced scaphoid nonunions.

## Introduction

A scaphoid fracture is a common injury of the wrist joint, accounting for 50%–80% of all carpal fractures (1). The unique anatomy of and tenuous blood supply to the scaphoid lead to its susceptibility to nonunion (2). Untreated scaphoid nonunions frequently involve dorsal intercalated segment instability (DISI) and humpback deformity (HD) and eventually lead to scaphoid nonunion advanced collapse (SNAC) (3, 4).

Conventional surgical treatment of displaced scaphoid fracture nonunions is typically performed by open reduction and internal fixation using a single compression screw with bone grafting. However, the single screw may not provide absolute stability. Slight motion can be detected at the fracture site, and sometimes, an additional or alternative method of fixation is used to augment the fracture fixation stability (5, 6). Biomechanical studies showed better bending and rotational stability by choosing two cannulated screw fixation than the single-screw construct (7–9).

There were only three previous studies that reported 34 scaphoid waist fracture nonunions (>6 months) with double-screw fixation (5, 10, 11). More than half of the fractures were fixed by antegrade screws using a dorsal approach. Although there is no conclusion that the palmar or dorsal approach is better than the other in scaphoid fracture surgery, a natural concern is that long-term arthritic change may result from making two large holes on the proximal scaphoid articular surface in the situation of two-screw fixation using the dorsal approach. This concern was the main reason we decided to choose the palmar approach and to insert the two cannulated screws in a retrograde fashion in the present study.

The aim of this study is to report on union rates, complication rates, and clinical outcomes of a consecutive single-surgeon series of patients treated with two retrograde cannulated headless compression screws with iliac bone grafting for scaphoid waist fracture nonunions with humpback deformities. We hypothesize that satisfactory outcomes could be obtained with the two-screw technique, with comparable or superior union rates to other techniques reported in the literature.

## Patients and methods

**Figure 1:**
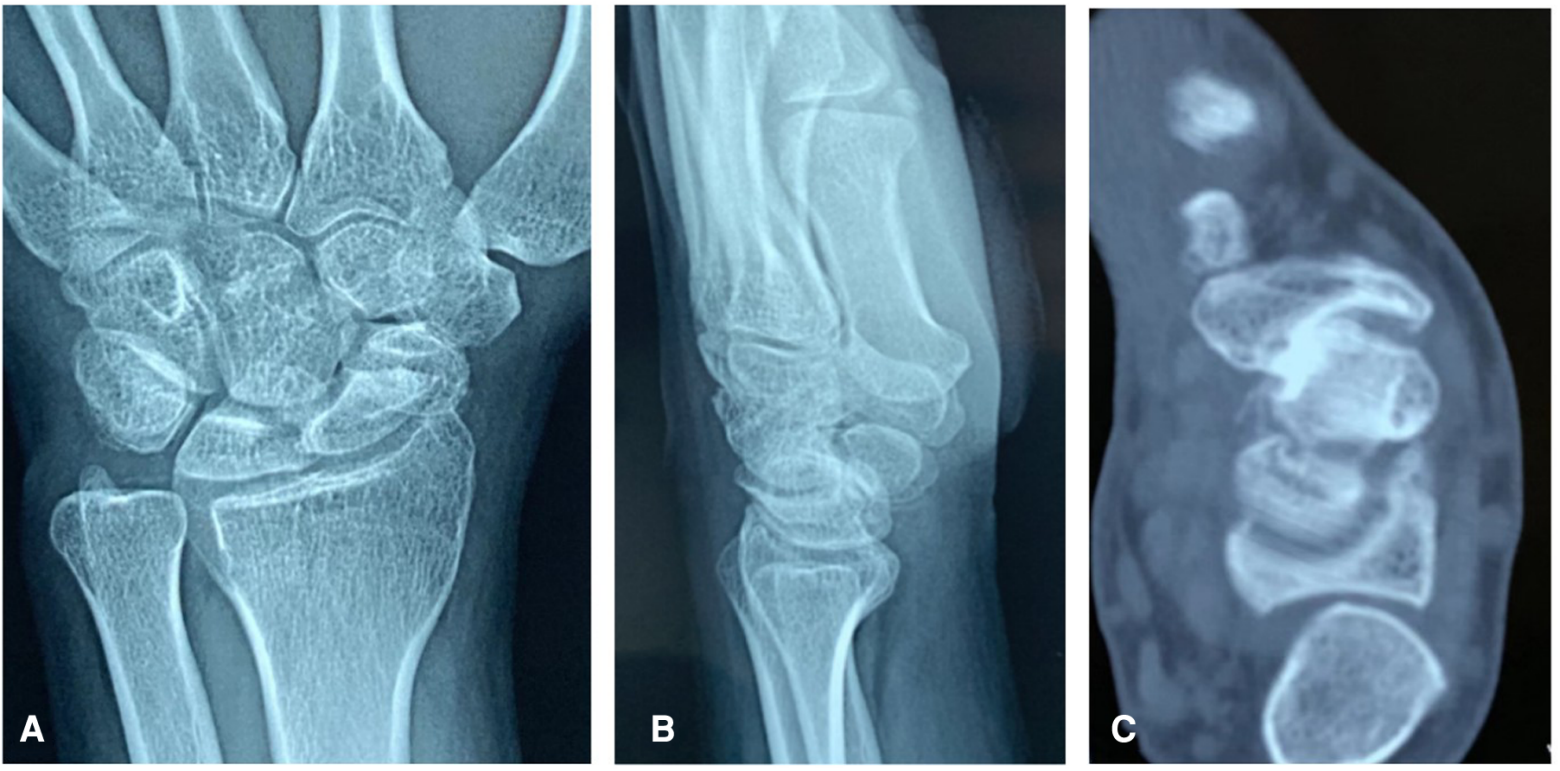
Preoperative anteroposterior (**A**) and lateral (**B**) x-ray and sagittal CT scan (**C**) of a scaphoid fracture nonunion with a humpback.

This retrospective study was approved by the ethics committee of our hospital prior to the commencement of any data collection. Medical records of scaphoid fracture nonunions with humpback deformities treated with open debridement, autologous iliac crest bone grafting, and internal fixation with two headless compression screws between January 2018 and December 2019 by a single surgeon were retrospectively reviewed. Inclusion criteria required an established scaphoid waist nonunion with humpback deformity suggested by preoperative computed tomography (CT) (Figure 1), without evidence of healing more than 6 months after injury and a minimum follow-up of 1 year. The lateral intrascaphoid angle (LISA), scapholunate angle (SLA), and height-to-length ratio (HLR) were examined as an index of displaced scaphoid nonunions (12). Exclusion criteria were (1) younger than 18 years of age, (2) additional injuries to the hand, (3) pregnancy, and (4) less than 1 year of follow-up.

Pertinent patient demographics (age, sex, smoking status) and injury specifics were included in the medical record review. Both preoperative and postoperative sagittal LISA and SLA were assessed and compared based on CT scans. Preoperative and postoperative grip strength (% of the healthy side), active range of motion (AROM), visual analogue scale (VAS), and patient-rated wrist evaluation (PRWE) scores at the final follow-up were obtained for all patients for comparison. Grip strength was measured by using a Jamar hand dynamometer and compared with the contralateral hand. AROM was measured before and after surgery using a goniometer. Available computed tomography scans were also carefully analyzed for cortical penetration of either screw. All complications were recorded. Whether the patient was able to perform previous work and sports activities comfortably was documented.

### Operative technique

**Figure 2:**
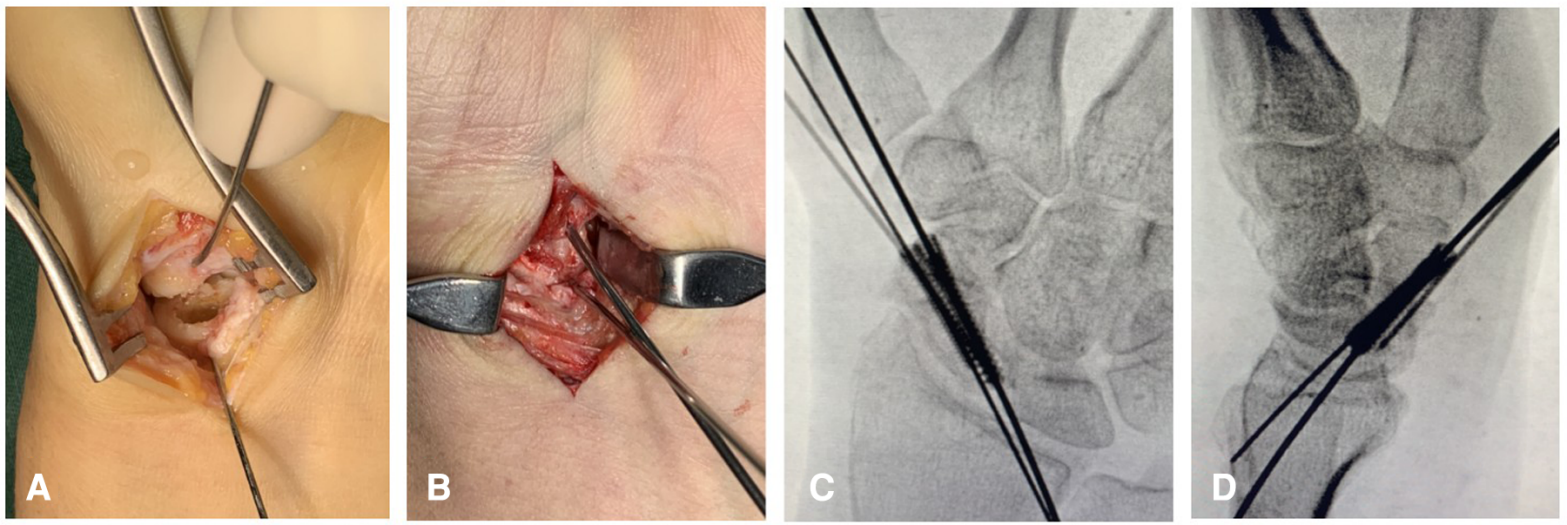
Fibrous tissue fragments were thoroughly debrided, leaving a shell of intact cartilage (**A**). Cancellous graft obtained from the patients’ iliac crest was impacted into the shell, and a wedge-shaped corticocancellous graft was inserted into the defect to maintain the reduction. Kirschner wires were used as joysticks to distract across the nonunion site (**B**). Two cannulated screws were inserted into the scaphoid along the guide wires, and the length and position of the screws were confirmed by x-ray (**C, D**).

The operation was performed under brachial plexus block anesthesia using an upper arm tourniquet. A straight incision was made in the distal forearm between the distal portion of the flexor carpi radialis (FCR) and the radial artery and was carried across the distal wrist crease using a hockey-stick incision that angles toward the base of the thumb. The FCR tendon was retracted ulnarly, and the radial artery was retracted radially. The wrist capsule was entered through a longitudinal incision from the volar lip of the radius to the proximal tubercle of the trapezium. The capsule and intracapsular ligaments were carefully divided and reflected sharply off the scaphoid with a scalpel. Fibrous tissue or pseudarthrosis of opposing bone surfaces of the proximal and distal fragments were thoroughly debrided by using a high-speed burr with constant irrigation, leaving a shell of intact cartilage (Figure 2A). We used Kirschner wires as joysticks to distract across the nonunion site. A cancellous graft obtained from the patient’s iliac crest was impacted into the shell, and a wedge-shaped cortico-cancellous graft was shaped to fit the gap between distal and proximal fragments and then inserted into the defect to maintain the reduction (Figure 2B).

The first guide wire was inserted along the long axis and was approximately 2 mm distant from the medial cortex of the scaphoid. Usually, two or three attempts were needed for guide wire insertion until the position of the first guide wire was ideal. Then, we inserted the second guide wire adjacent to the first, but separated in the radioulnar plane by the distance of the diameter of the planned screws. Usually, one or two attempts were needed for second screw guide wire insertion.

After the positions of the guide wires were verified by the C-arm, two cannulated screws (one Acutrak Mini and one Acutrack Mirco Hillsboro, OR, USA) were inserted into the scaphoid by the palmar approach. For most cases, we inserted the 2.5 mm-diameter screw through the radial guide wire first and then the 3.5 mm-diameter screw through the ulnar guide because the bone quality of the ulnar side of the scaphoid waist was the highest. Only in one female patient, we used two 2.5 mm-diameter screws, while in two male patients, we inserted two 3.5 mm-diameter screws into the scaphoid. The length and position of the screws were confirmed by C-arm (Figures 2C,D). Finally, the wires were removed, and the capsule and radioscaphocapitate ligament were repaired. All procedures were performed by one senior surgeon.

### Postoperative management and follow-up

**Figure 3:**
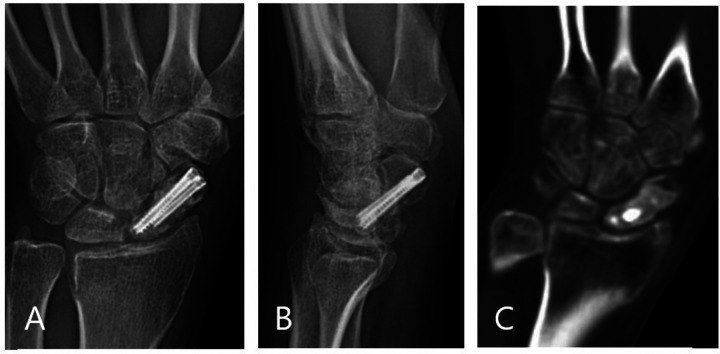
Anteroposterior (**A**) and lateral (**B**) x-ray and coronal CT scan (**C**) confirming the solid union of the fracture site.

All patients were allowed to gently move the wrist on the first day postoperatively, as tolerated, without cast immobilization. Standard radiographs of the scaphoid (scaphoid, posteroanterior, lateral, and semi-pronated oblique views) were obtained monthly. When a union was considered, we used x-ray and CT scans to confirm the solid union of the fracture site (Figure 3). Noncontact sports were resumed gradually after the fracture union was confirmed. We measured the SLA, LISA, and HLR before and after surgery in each patient to evaluate carpal alignment. Clinical assessments were performed at 1, 2, 3, and 6 months and thereafter annually.

### Statistical analysis

Data that were normally distributed were described using means and SDs. Nonparametric data were described using medians, minimum and maximum values, and interquartile ranges. Differences in pre- and post-operative data were identified using paired Student’s *t*-tests for normally distributed data and Wilcoxon’s tests for nonparametric data. To compare the operated wrist with the unaffected, contralateral wrist, dependent Student’s *t*-tests were used. A *p*-value less than 0.05 was considered statistically significant. Statistical analyses were conducted using SPSS version 25.0 (IBM Corp., Armonk, NY).

## Results

Eighteen patients were men and three patients were women, aged 18–46 (mean 29.8) years. The average time from injury to surgery was 38.3 (12–250) months. There was humpback deformity in eight patients and scaphoid nonunion advanced collapse (SNAC) in 13 patients (stage I in 10 and stage II in 3 patients), which included one patient with avascular necrosis (AVN).

Bone union was achieved in all cases, with a follow-up of 24–48 (mean 30.5) months. The average healing time was 2.7 months (range, 2–4 months) after surgery, and 14 scaphoids of 21 (66.7%) patients healed by 8 weeks.

At the time of the final follow-up, there was a statistically significant improvement in AROM, grip strength, VAS, and PRWE scores compared with the preoperative values (Table 1). There were also significant improvements in radiographic parameters for assessing humpback deformity compared with preoperative values (Table 2).

**Table 1:** Clinical outcomes of pre- and post-operation.

	Preoperative	Postoperative	*p*-value
AROM (°)	76 ± 52	118 ± 46	0.028
Grip strength (% of healthy side)	50 ± 23	83 ± 26	0.033
VAS	7 ± 1	3 ± 1	0.018
PRWE	56 ± 30	14 ± 11	0.025

AROM, active range of motion; RPWE, patient-rated wrist evaluation.

**Table 2:** Preoperative and postoperative radiographic data of patients with humpback deformities.

	Preoperative	Postoperative	*p*-value
SLA (°)	71 ± 14	50 ± 13	0.032
LISA (°)	56 ± 6	32 ± 7	0.028
HLR	69 ± 8	50 ± 12	0.016

SLA, scapholunate angle; LISA, lateral intrascaphoid angle; HLR, height-to-length ratio.

No patient reported any major complications, and two patients complained of slight discomfort at the incisional scar. CT scans showed no evidence of cortical penetration of either screw, in particular, at the scaphocapitate joint in all patients. All patients returned to previous work except three patients who were not at full capacity. No case required a second procedure for revision surgery or hardware removal.

## Discussion

Scaphoid fracture nonunions have been challenging for hand surgeons for the last two decades, especially those with established displacement. Some surgeons reported cases without bone grafting that can achieve bone union with screw fixation or staple alone (13). De Vitis et al. reported using gelled platelet-rich plasma (GPRP) instead of bone grafting during the fixation of scaphoid fractures (14, 15). However, the types of scaphoid fracture nonunions that can achieve bone union without bone grafting are still unclear. Some studies demonstrated that only linear-type and well-aligned fractures could achieve bone union without bone grafting (13, 16). The current standard treatment is open reduction and bone grafting with fixation options including single-screw, double-screw, and plate fixation. Some surgeons recommended vascularized bone grafting for a higher union rate and earlier union (17). On the other hand, Zhang et al. compared pedicled vascularized and nonvascularized bone grafting and concluded no difference in functional recovery (18). In addition, the shape of the bone graft is important, and a wedge bone graft is commonly used. De Vitis et al. reported 42 cases of the modified Matti–Russe technique of grafting for treating scaphoid nonunions with good results (19), and Dustmann et al. reported 52 cases with a high healing rate (20). However, in other studies, no difference was observed between different bone grafts (21, 22). Many surgeons believe that fixation stability may be an important requisite for healing in the management of scaphoid nonunions (23–25). These surgeons also pointed out that most scaphoid nonunions still have enough healing potential if the biomechanical strain on the fracture site is reduced. Therefore, we suggest that fixation stability is more important than the choice of bone graft.

However, a single screw may not be able to provide considerable stability, particularly torsional stability, in a scaphoid with segmental bone loss. Recent biomechanical studies demonstrated that double-screw or plate fixation showed greater stability, stiffness, and energy absorption, especially rotational stability, than a single compression screw (7–9). Clinically, some surgeons advocate using a derotational Kirschner wire in addition to single-screw fixation to enhance torsional stability in acute or chronic scaphoid fractures (6, 26, 27). In Diaz's randomized controlled trial, sometimes an additional fixation is needed to augment the strength of the single-screw construct, and some mobility can be detected even after screw fixation. Therefore, a postoperative protocol with single-screw fixation often includes some sort of temporary immobilization, by either bandage, splint, or cast, for at least 2 weeks. With double-screw fixation, we believe that more rigid fixation allows partial loading of the wrist immediately after surgery, and our postoperative regimen allows patients to bear some weight on the wrist, as tolerated.

In the past 10 years, numerous papers have reported using two headless compression screws or scaphoid locking plates with a variety of nonvascularized bone grafting techniques in patients with unstable scaphoid nonunions, and even in the nonunions with avascular necrosis of the proximal pole (28). The union rate of these series was comparable to or better than that achieved in other series (22, 29–31). The authors believe that important factors in ensuring the healing rate of a scaphoid nonunion were sufficient structural integrity and rotational control of the fixation. Compared with double-screw fixation, locking plate fixation appears to be associated with a greater risk of complications, mainly plate impingement to the distal radius, resulting in plates requiring removal more frequently than headless compression screws (32). Thus, some surgeons proposed that the plate fixation method serves as an additional option in the treatment of recalcitrant scaphoid nonunions or patients with failed prior surgical intervention (33). With double-screw fixation, we believe that more rigid fixation allows partial loading of the wrist immediately after surgery, and our postoperative regimen allows patients to bear some weight on the wrist, as tolerated.

In the present study, bony union was achieved in all patients, with a follow-up of at least 2 years. The average healing time was 2.5 months, and 75% of patients healed by 8 weeks. In the literature, reported union rates after headless compression screw and bone grafting have varied from 71% to 100%, and the mean period for bone union has varied from 3.5 months to 5 months (34–37). Among these studies, only three have reported using double-screw fixation for scaphoid fracture nonunions (5, 10, 11). Garcia et al. reported a 100% healing rate for double-screw fixation in 19 patients with different fracture locations, with vascularized or nonvascularized bone grafting by volar or dorsal approaches, and by using different types of market-available screws. An external bone stimulator was used immediately postoperatively in 12 patients and was continued for 4–6 weeks (5). Eugene et al. reported 21 delayed union or nonunion (>6 months) scaphoid waist fractures using antegrade double-screw fixation by the dorsal approach with cancellous distal radius bone grafts. Of these, 10 cases were nonunions with a healing rate of 90% (10). In our study, we present homogeneous scaphoid waist fracture nonunions with humpback deformity treated by retrograde double-screw fixation using a palmar approach with a combination of cancellous and corticocancellous iliac crest bone grafts. All fractures healed within 4 months, and all humpback deformities were corrected.

Some authors proposed that using a volar or dorsal approach are the surgeon's preference since both approaches have their advantages and disadvantages. No clear superiority of one approach has been identified over the other (38–40). However, all of the above debates were based on the single-screw fixation technique. For the situation of double-screw insertion by the dorsal approach, the radioscaphoid joint, violated by the drill, raises the issue of whether drilling twice into this known load-bearing area is a good idea. Furthermore, it is unknown whether long-term arthritic change may result from making two large holes in the proximal scaphoid articular surface. These natural concerns led us to choose retrograde screw insertion by the palmar approach. Quadlbauer et al. also reported retrograde double-screw fixation in 12 cases of scaphoid waist nonunions in their comparative study (11). The authors compared single- vs. double-screw fixation vs. plate fixation with iliac crest bone grafting in 42 patients, with or without extracorporeal shockwave therapy. The final bony healing rate was 60% in single-screw fixation and 83% in double-screw fixation, which was much lower than the healing rate reported by large-scale systematic reviews (41, 42).

The placement of the screws within the scaphoid is important, and it is often difficult to achieve optimal screw placement. Garcia et al. suggested that, when placing two screws, they should be parallel to the long axis of the scaphoid and not directly adjacent to each other in the coronal plane (5). They should also be separated, at minimum, by the diameter of the screws to be inserted. However, it is currently unclear which is the best orientation to arrange the two screws, for example, in the anteroposterior plane or in the radioulnar plane. Lee et al. suggested that screw placement should be based on regional bone strength (43). The authors conducted a regional analysis of the bony microarchitecture of the scaphoid, measuring bone density and bone quality, to give an overall indication of bone strength. The authors found that the axis running from the ulnar side of the distal tuberosity to the radial side of the proximal pole, in addition to the ulnar side of the scaphoid waist, exhibited the highest bone strength parameters, based on the osseous microarchitecture. From this analysis, it was proposed that screws should be placed through these regions to achieve maximal strength of fixation. In this current series, all screws were inserted in an antegrade fashion, with the goal of screw placement to be along the longitudinal axis of the scaphoid, maximally separated in the coronal plane. However, further biomechanical studies are required to test whether this is the optimum position of screw placement.

Central screw placement is technically demanding, even for experienced surgeons. One may imagine that placing two screws is more challenging than placing a single screw centrally. The robotic-assisted scaphoid double-screw insertion technique, as we described previously, may provide a practical solution to reduce surgery time and improve guide wire insertion accuracy (44). This novel technique has not been accepted in treating displaced nonunions with open debridement and bone grafting. However, Garcia's experience is that placing two screws off the central axis is actually simpler than placing a single, centrally positioned screw (5). In fact, it is not possible or even necessary to insert either of the screws in a central position in the technique of double-screw fixation. There is some debate on the best orientation to arrange the two parallel screws, in the coronal or sagittal plane. Quadlbauer also reported two screws not parallel to each other (11). Since no biomechanical studies exist to explore the best fashion to arrange the position of the two screws, we aimed to insert two parallel screws along the longitudinal axis, as we defined previously, separated by the radioulnar plane (45). We always chose a 3.5 mm-diameter screw (except for one female patient) for the ulnar side screw to maximize the strength of the construct because the ulnar side of the scaphoid waist had the highest bone quality parameters, based on a bony microarchitecture study (43). Our experience is that if one surgeon can manage to precisely insert a single screw into the desired central position within three guide-wire attempts, it is not that difficult to insert two parallel screws in the scaphoid without cortical penetration.

This study had some limitations, including the absence of a control group and the small sample size. In the future, multicenter prospective randomized control trials are needed to verify the positive effects and higher union rate obtained by double-screw fixation than single-screw fixation for displaced scaphoid fracture nonunions. Further studies should also be carried out to investigate how to insert two screws to achieve the best fixation strength when using the double-screw fixation technique.

## Data Availability

The raw data supporting the conclusions of this article will be made available by the authors without undue reservation.
